# Risk factors and evaluation of keratoconus progression after penetrating keratoplasty with anterior segment optical coherence tomography

**DOI:** 10.1038/s41598-020-75412-y

**Published:** 2020-10-29

**Authors:** Junko Yoshida, Tetsuya Toyono, Rika Shirakawa, Takashi Miyai, Tomohiko Usui

**Affiliations:** 1 Department of Ophthalmology, The University of Tokyo Hospital, 7-3-1 Hongo, Bunkyo-ku, Tokyo, 113-8655 Japan; 2 Department of Ophthalmology, The International University of Health and Welfare, 1-4-3, Mita, Minato-ku, Tokyo, 108-8329 Japan

**Keywords:** Diseases, Medical research, Risk factors

## Abstract

To determine the risk factors and unique characteristics of keratoconus (KC) progression after penetrating keratoplasty (PK), anterior segment optical coherence tomography parameters were statistically analyzed in comparison with eyes undergoing PK for other diseases as a control. Ninety-one eyes maintaining clear PK grafts for over 10 years were divided into 2 groups according to the primary indication for PK (KC vs Others groups). Corneal thinning indicators (inferior host thinnest corneal thickness/central corneal thickness [IHT/CCT], inferior graft thinnest corneal thickness/CCT [IGT/CCT]), were smaller whereas anterior chamber depth, and steepest corneal power (Ks), and maximum corneal power (K_max_) were larger in the KC group with statistical significance. Graft size, K_max_ and Ks correlated with IHT/CCT and IGT/CCT in the KC group. These correlations were not detected in controls. Graft size and postoperative period were selected by multivariate regression analysis as factors for corneal ectatic changes in the KC group. In conclusion, KC eyes long after PK show inferior graft and host corneal thinning, and corneal protrusion. Corneal power parameters such as K_max_ or Ks can be used to monitor KC progression after PK. A small graft might lead to KC progression after PK.

## Introduction

Keratoconus (KC) is a bilateral, progressive, and non-inflammatory corneal ectatic disease whose clinical characteristics are paracentral corneal thinning and corneal protrusion, mainly occurring in juveniles^1,2^. The KC prevalence in the latest report was higher (1:375) than expected based on previous reports, probably a result of advances in imaging techniques^3^. According to the US Eye Bank statistical report in 2017, ~ 38,000 penetrating keratoplasties (PK) are performed annually, primarily for KC, which accounts for ~ 25% of the indication-known PK cases. The prognosis of PK for KC eyes is excellent, with an over 90% 10-year graft survival rate^4–6^. KC sometimes recurs in the long-term—usually decades—after PK. As there are few reports^7–10^ on recurrent KC, the details of recurrence, such as the frequency and characteristics, remain unknown.

We recently reported that KC recurs in up to 36% of PK patients who had maintained clear grafts for over 10 years^8^. Also, in a report of acute hydrops cases in recurrent KC^7^, Descemet’s membrane breaks are seen around the graft-host junction, not around the center as usually seen in keratoplasty-naïve KC. The characteristics are unsurprisingly different between recurrent and keratoplasty-naïve KCs. Recurrent KC might thus not be a rare condition in long-surviving grafts. In addition, as the efficacy of corneal collagen crosslinking to prevent KC progression was recently recognized worldwide^11–17^, corneal collagen crosslinking might need to be considered for recurrent KCs^18^. In that case, proper evaluation of KC progression after PK is necessary, and it is important to clarify the characteristics and diagnostic parameters. The problem here, however, is that there are no clear and objective diagnosing criteria of recurrent KC and thus, whole aspect of KC progression after PK is still unclear.

The present study aimed to determine the objective characteristics of KC progression after PK, such as corneal thinning or ectatic changes, using anterior segment optical coherence tomography (AS-OCT), in comparison with eyes undergoing PK eyes for other diseases as a control. We also explored the risk factors for KC progression after PK. A retrospective comparative analysis of post-PK eyes with a greater than 10-year follow-up period was performed.

## Results

**Figure 1:**
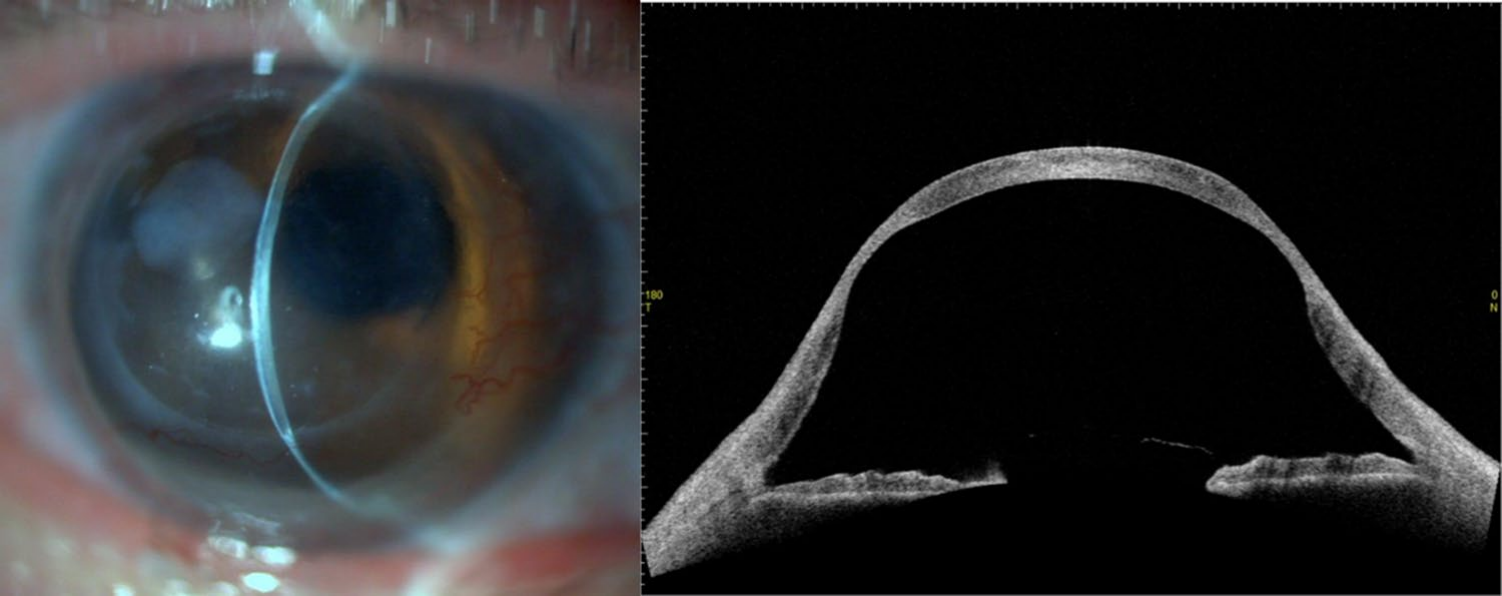
Representative images of an eye of 65-year-old man who underwent penetrating keratoplasty for keratoconus 36 years ago. The corneal thinning in the mid-peripheral area and corneal protrusion are remarkable.

Representative images of KC recurrence are shown in Fig. 1. The patient characteristics and parameters measured by AS-OCT are shown in Table 1. The KC group comprised 46 eyes and the Others group comprised 45 eyes. Postoperative periods (approximately 25 years) did not differ between the 2 groups. Mean graft size was 7.0 mm in the KC group and 7.3 mm in Others group (*p* = 0.042). Anterior chamber depth (ACD) was 3.67 mm in KC group and 2.95 mm in Others group (*p* < 0.0001). The inferior corneal thinning indicators (inferior host thinnest corneal thickness/central corneal thickness [IHT/CCT] and inferior graft thinnest corneal thickness/CCT [IGT/CCT]) were smaller in the KC group (*p* = 0.00034 and *p* = 0.025, respectively). Keratometric values, such as the steepest corneal power (Ks), average corneal power (AvgK) and maximum keratometric corneal power (K_max_), were significantly larger in the KC group.

**Table 1:** Patient characteristics and measured parameters in the KC and Others groups.

	KC (N = 46)		Others (N = 45)		*p*
	Mean	SD	Mean	SD	
Age at PK (years)	29.5	9.4	46.1	18.1	< 0.0001
Postoperative period (years)	27.7	8.6	25.5	10.5	0.27
Current age (years)	57.2	10.3	71.6	15.5	< 0.0001
Man to woman ratio (%)	76.1	–	44.4	–	0.0027
Graft size (mm)	7.0	0.69	7.3	0.61	0.042
ACD (mm)	3.67	0.72	2.95	0.63	< 0.0001
CCT (µm)	570	58.1	562	68.0	0.52
IHT (µm)	509	165.7	624	119.0	0.00027
IGT (µm)	585	91.0	618	96.3	0.093
IHT/CCT	0.90	0.32	1.12	0.23	0.00034
IGT/CCT	1.03	0.16	1.11	0.17	0.025
Ks (D)	57.5	8.8	51.5	6.5	0.00035
AvgK (D)	53.2	7.5	48.2	5.9	0.00061
CYL (D)	8.5	6.3	6.5	4.3	0.077
**Fourier analysis (D)**					
Spherical	52.9	7.4	48.1	5.9	0.00089
Regular astigmatism	4.4	3.1	3.4	2.2	0.083
Asymmetry	4.0	4.3	3.4	2.5	0.38
HOI	0.85	0.50	1.02	1.03	0.32
K_**max**_ (D)	66.9	14.5	59.5	11.0	0.012

*KC* keratoconus, *PK* penetrating keratoplasty, *ACD* anterior chamber depth, *CCT* central corneal thickness, *IHT* inferior thinnest host cornea thickness, *IGT* inferior thinnest graft thickness, *IHT/CCT* IHT ratio to CCT, *IGT/CCT* IGT ratio to CCT, *Ks* steepest corneal power, *D* diopters, *AvgK* average corneal power, *CYL* cylinder, *HOI* higher order irregularity, *K*_*max*_ Maximum corneal power.

**Figure 2:**
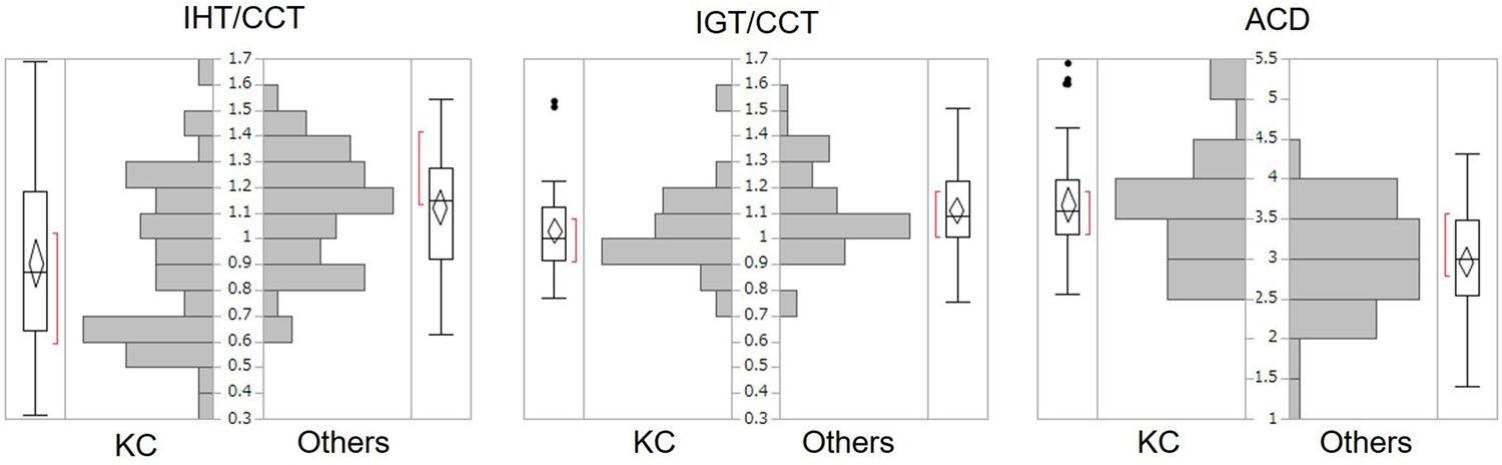
Distributions of corneal ectasia indicators (IHT/CCT, IGT/CCT, and ACD) in the KC and Others groups. Both the inferior host cornea and graft were thinner in the KC group, but the tendency was more obvious in the host cornea. The ACD tend to be larger in the KC group, suggesting more severe corneal protrusion.

Figure 2 shows the distributions of the corneal ectasia indicators (IHT/CCT, IGT/CCT, and ACD) in each group. IHT/CCT peaked at around 0.6–0.7 in the KC group and at 1.1–1.2 in the Others group. IGT/CCT peaked at 0.9–1.0 in the KC group and at 1.0–1.1 in the Others group.

**Figure 3:**
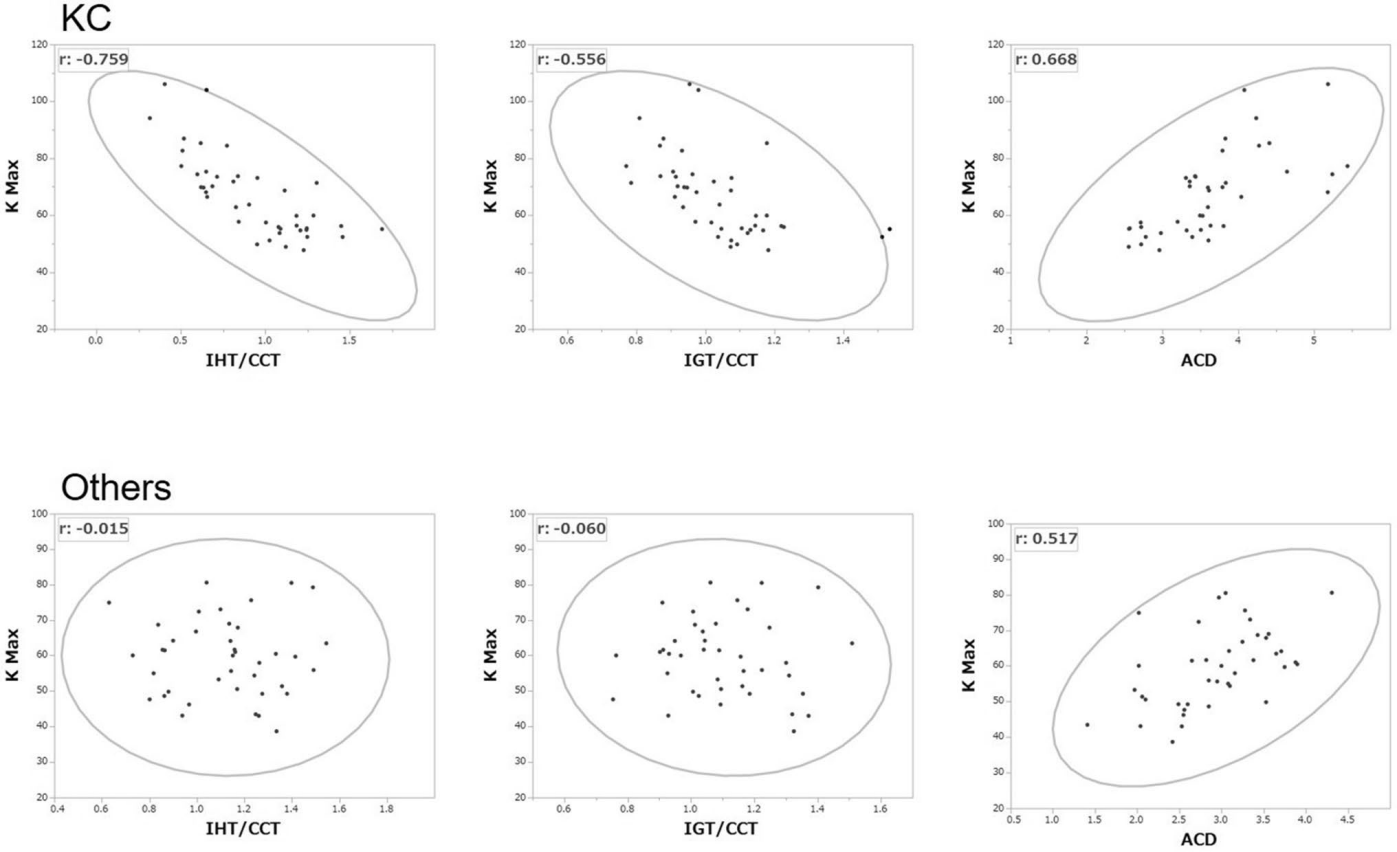
Scatterplots of K_max_ and the corneal ectasia indicators (IHT/CCT, IGT/CCT, and ACD) in the KC (upper graphs) and Others (lower graphs) groups. K_max_ was strongly correlated with host and graft thinning in the KC group but not in the Others group. Deeper ACD correlated with a larger K_max_ in both groups. Density ellipses show p = 0.990.

Correlation coefficients in each group are shown in Table 2. In the KC group, graft size was strongly correlated with the corneal thinning indicators (IHT/CCT and IGT/CCT) and keratometric parameters, but this tendency was not observed in the Others group. IHT/CCT and IGT/CCT were correlated with most of the keratometric parameters in the KC group. In the Others group, IHT/CCT and IGT/CCT were correlated with age, but this was not observed in the KC group. Figure 3 shows scatterplots between K_max_ and the corneal ectasia indicators. A negative correlation was detected between K_max_ and IHT/CCT or IGT/CCT in the KC group, but not in the Others group. Both groups showed a tendency toward a positive correlation between K_max_ and ACD.

**Table 2:** Correlation of each parameter in the KC and others groups: Pearson correlation coefficients.

	Age at PK	Postop period	Graft size	ACD	IHT/CCT	IGT/CCT	Ks	AvgK	CYL	Fourier analysis				K_max_
										Sph	Reg	Asym	HOI	
**KC group**														
Current age	0.628	0.517	− 0.249	0.005	− 0.040	0.061	0.167	0.096	0.238	0.075	0.259	0.217	0.354	0.055
*p*	< 0.0001	0.001	0.121	0.975	0.809	0.710	0.304	0.556	0.138	0.646	0.106	0.178	0.025	0.735
Age at PK		− 0.342	0.081	0.108	− 0.113	0.026	0.214	0.147	0.251	0.132	0.291	0.227	0.402	0.157
*p*		0.031	0.621	0.507	0.487	0.873	0.184	0.366	0.118	0.418	0.069	0.159	0.010	0.334
Postop period			− 0.389	− 0.120	0.077	0.044	− 0.035	− 0.046	0.012	− 0.054	− 0.007	0.013	− 0.015	− 0.103
p			0.013	0.462	0.637	0.785	0.833	0.779	0.942	0.739	0.968	0.938	0.927	0.526
Graft size				− 0.265	0.503	0.564	− 0.417	− 0.439	− 0.124	− 0.439	− 0.135	− 0.339	− 0.368	− 0.434
*p*				0.098	0.001	< 0.0001	0.007	0.005	0.447	0.005	0.406	0.032	0.019	0.005
ACD					− 0.622	− 0.438	0.543	0.603	0.073	0.608	0.120	0.368	0.359	0.668
*p*					< 0.0001	0.005	0.0003	< 0.0001	0.656	< 0.0001	0.460	0.020	0.023	< 0.0001
IHT/CCT						0.754	− 0.696	− 0.658	− 0.383	− 0.645	− 0.427	− 0.576	− 0.523	− 0.759
*p*						< 0.0001	< 0.0001	< 0.0001	0.015	< 0.0001	0.006	0.0001	0.001	< 0.0001
IGT/CCT							− 0.530	− 0.534	− 0.212	− 0.531	− 0.222	− 0.401	− 0.327	− 0.556
*p*							0.0004	0.0004	0.189	0.0004	0.168	0.010	0.040	0.0002
**Others group**														
Current age	0.815	0.069	0.044	− 0.059	0.275	0.183	0.093	0.112	− 0.029	0.085	− 0.101	0.200	− 0.037	0.028
*p*	< 0.0001	0.674	0.786	0.718	0.086	0.259	0.570	0.490	0.861	0.604	0.535	0.216	0.819	0.862
Age at PK		− 0.522	0.143	0.150	0.423	0.275	0.249	0.243	0.084	0.223	0.045	0.382	0.064	0.265
*P*		0.001	0.377	0.354	0.006	0.086	0.121	0.130	0.605	0.167	0.784	0.015	0.695	0.099
Postop period			− 0.182	− 0.346	− 0.325	− 0.205	− 0.293	− 0.253	− 0.187	− 0.259	− 0.226	− 0.363	− 0.165	− 0.424
*p*			0.261	0.029	0.041	0.205	0.067	0.115	0.248	0.106	0.162	0.021	0.308	0.006
Graft size				0.355	0.260	− 0.007	0.037	0.012	0.078	0.028	0.128	− 0.022	0.099	0.149
*p*				0.025	0.106	0.965	0.820	0.941	0.632	0.863	0.430	0.895	0.543	0.359
ACD					0.089	− 0.017	0.316	0.321	0.075	0.360	0.155	0.162	0.332	0.517
*p*					0.587	0.915	0.047	0.044	0.645	0.023	0.338	0.319	0.036	0.001
IHT/CCT						0.752	− 0.078	− 0.088	0.007	− 0.094	0.009	0.151	− 0.047	− 0.015
*p*						< 0.0001	0.633	0.587	0.965	0.564	0.958	0.351	0.775	0.929
IGT/CCT							− 0.142	− 0.167	0.028	− 0.175	0.021	0.234	− 0.041	− 0.060
*p*							0.381	0.302	0.864	0.279	0.896	0.146	0.800	0.711

Underline values indicate *p* < 0.05.

*KC* keratoconus, *PK* penetrating keratoplasty, *Postop period* postoperative period, *ACD* anterior chamber depth, *IHT* inferior thinnest host cornea thickness, *CCT* central corneal thickness, *IGT* inferior thinnest graft thickness, *IHT/CCT* IHT ratio to CCT, *IGT/CCT* IGT ratio to CCT, *Ks* steepest corneal power, *AveK* average corneal power, *CYL* cylinder, *Sph* spherical, *Reg* regular astigmatism, *Asym* asymmetry, *HOI* higher order irregularity, *K*_*max*_ maximum corneal power.

Table 3 shows the results of the multivariant regression analysis with the corneal ectasia indicators as dependent variables and the patient background characteristics as independent variables, aiming to determine the risk factors for corneal ectatic changes. Graft size was selected as explaining the corneal thinning indicators with negative estimates in the KC group, but not in the Others group. Graft size was also selected as explaining ACD in both groups, negatively in the KC group and positively in the Others group. Table 4 shows the results of the multivariant regression analysis to determine which corneal ectasia indicators were associated with corneal powers and astigmatisms. In the KC group, IHT/CCT was selected as explaining all of the keratometric parameters, but this was not the case in the Others group.

**Table 3:** Factors associated with corneal ectasia indicators in KC and others: multivariant regression analysis of IHT/CCT, IGT/CCT, and ACD as the dependent variables with the independent variables of age at PK, current age, postoperative period, sex, and graft size.

Dependent	KC			Others		
	Selected	Estimates	*p*	Selected	Estimates	*p*
IHT/CCT	Postoperative period	0.012	0.021	Age at PK	0.005	0.004
	Graft size	0.286	< 0.0001			
IGT/CCT	Postoperative period	0.006	0.019	Age at PK	0.003	0.067
	Graft size	0.155	< 0.0001			
ACD	Postoperative period	− 0.026	0.075	Postoperative period	− 0.017	0.044
	Graft size	− 0.418	0.019	Graft size	0.310	0.036

*KC* keratoconus, *IHT* inferior thinnest host cornea thickness, *CCT* central corneal thickness, *IGT* inferior thinnest graft thickness, *IHT/CCT* IHT ratio to CCT, *IGT/CCT* IGT ratio to CCT, *ACD* Anterior chamber depth, *PK* Penetrating keratoplasty.

**Table 4:** Factors associated with corneal powers and astigmatism in the KC and Others groups: Multivariant regression analysis on AS-OCT parameters as dependent variables with the independent variables IHT/CCT, IGT/CCT and ACD.

Dependent	KC			Others		
	Selected	Estimates	*p*	Selected	Estimates	*p*
Ks	IHT/CCT	− 20.61	< 0.0001	ACD	3.25	0.034
AvgK	IHT/CCT	− 11.98	0.001	ACD	2.99	0.032
	ACD	3.17	0.036			
CYL	IHT/CCT	− 7.77	0.013	N/A	–	–
Fourier Sph	IHT/CCT	− 11.22	0.003	ACD	3.36	0.015
	ACD	3.38	0.026			
Fourier Reg	IHT/CCT	− 4.39	0.005	N/A	–	–
Fourier Asym	IHT/CCT	− 8.41	< 0.0001	IGT/CCT	3.54	0.122
Fourier HOI	IHT/CCT	− 0.88	0.0003	ACD	0.54	0.026
K_max_	IHT/CCT	− 28.30	< 0.0001	ACD	8.90	0.001
	ACD	6.05	0.016			

*KC* keratoconus, *AS-OCT* anterior segment optical coherence tomography, *IHT* inferior thinnest host cornea thickness, *CCT* central corneal thickness, *IGT* inferior thinnest graft thickness, *IHT/CCT* IHT ratio to CCT, *IGT/CCT* IGT ratio to CCT, *ACD* anterior chamber depth, *Ks* steepest corneal power, *AvgK* average corneal power, *CYL* cylinder, *Sph* spherical, *Reg* regular astigmatism, *Asym* asymmetry, *HOI* higher order irregularity, *K*_*max*_ maximum corneal power.

## Discussion

In this study, we performed a comparative analysis of patients who underwent PK for KC and other diseases as a control to determine the characteristics and risk factors of KC progression after PK using AS-OCT data. The mean postoperative period was over 25 years, which is sufficiently long to determine the corneal ectatic change. To quantify corneal thinning, we calculated corneal thinning indicators: IHT/CCT and IGT/CCT, each of which explains the relative thickness in the inferior host cornea and inferior graft cornea, respectively. Also, we used ACD as a corneal protrusion parameter. These three parameters were set to evaluate corneal ectasia in this study.

Comparing the KC and Others groups, KC had a deeper ACD, and smaller IHT/CCT and IGT/CCT. The normal cornea is thinnest at the center and gradually becomes thicker nearer the peripheral area. An IHT/CCT or IGT/CCT of less than or nearly 1.0 indicates inferior corneal thinning. The peak IHT/CCT was around 0.6–0.7 in the KC group and around 1.1–1.2 in the Others group (Fig. 2). Likewise, the normal ACD should be ~ 3.0 mm, which was indeed observed in the Others group, but the peak of the KC group fell to around 3.5–4.0, suggesting corneal protrusion in KC eyes. Those results suggest KC eyes showed a corneal ectatic change. This corneal ectatic change is also supported by the larger values of keratometric parameters such as Ks, AvgK, Sph, and K_max_. In contrast, astigmatic parameters such as cylinder (CYL), regular astigmatism (Reg), asymmetry (Asym), or higher order irregularity (HOI) did not differ between the groups, probably because of the nature of PK, which causes strong astigmatism. Thus, in post-PK eyes, astigmatic change should not be important for evaluating KC progression, although astigmatism is usually important for judging progression in keratoplasty-naïve KC eyes.

Pearson correlation coefficients revealed interesting differences between the KC and Others groups. First, positive correlations between the graft size and corneal thinning parameters observed in the KC group were not observed in the Others group. Graft size was also negatively correlated with keratometric parameters in the KC group, but not in the Others group. In other words, a smaller graft may cause more thinning and more severe protrusion, i.e., KC progression. Second, negative correlations between corneal thinning parameters and keratometric parameters were observed in the KC group, but not in the Others group. This suggests that inferior corneal thinning is related to stronger keratometric values, and thus those parameters are reliable for evaluating KC progression after PK. Positive correlations between ACD and keratometic parameters were common to both groups, indicating that eyes with a deeper ACD tend to have a stronger corneal power regardless of the primary indications for PK. This supports the rationale for using ACD as a corneal protrusion parameter, although ACD sometimes depends on cataract formation.

As described above, the corneal ectasia parameters that we set (IHT/CCT, IGT/CCT, and ACD) can probably be used as indicators of KC progression after PK. Next, we performed multivariant regression analysis to determine the risk factors for KC progression. From the examined candidate risk factors (age at PK, current age, postoperative period, sex, and graft size), postoperative period and graft size were selected. In other words, the KC could progress over time after PK and a smaller graft might lead to a more severe progression. The fact that the postoperative period was selected is reasonable given the progressive nature of KC. As for the reason graft size was selected, we speculate that a small graft might be inadequate for removing all the affected KC lesion. Thus, surgeons who perform PK in KC eyes should be careful to choose an adequately sized graft.

Whether it is the host cornea or the graft that develops KC progression has remained controversial. The finding that both IHT/CCT and IGT/CCT were smaller in the KC group than in the Others group supports the notion that the progression occurs in both the graft and host. This notion is also supported by our previous reports on histopathologic specimens of corneas with recurrent KC^7,8^. In the regression analysis that we performed to find out the factors contributing to the keratometric changes, IHT/CCT, a parameter reflecting host thinning, was selected in the analyses. In contrast, IGT/CCT, reflecting graft thinning, was not selected. This means that keratometric changes occurred mainly because of changes to the host cornea.

A limitation of the study is that we did not evaluate the lens status, e.g., phakic or pseudophakic, which could affect the ACD, a protrusion parameter. As the ACD correlated with the corneal steepening in both groups, however, the effect of lens status was likely minimal. Another limitation is that we did not show the data at the time of PK. However, there should not have been big differences in tomographic data between the groups just after PK. For example, KC eyes tend to have deeper ACD, but postoperative normalization of ACD by PK has been reported^19,20^. Also, inferior graft thinning, which was observed only in KC group, should not have existed at the time of PK.

In conclusion, KC eyes long after PK tend to show inferior graft and host corneal thinning, and corneal protrusion, suggesting KC progression. Corneal power parameters such as the K_max_ or Ks can be used to evaluate KC progression after PK, but astigmatic parameters such as CYL cannot. Although both host and graft showed KC changes, keratometric changes mainly resulted from host corneal thinning. When performing PK in KC patients, it is important to select an adequately sized graft.

## Methods

The study was approved by the ethics committee of the University of Tokyo Hospital. Requirements of informed consent were waived for this retrospective chart review by the ethics committee of the University of Tokyo Hospital. Data analysis was performed on de-identified data, in accordance with the relevant guidelines and regulations.

**Figure 4:**
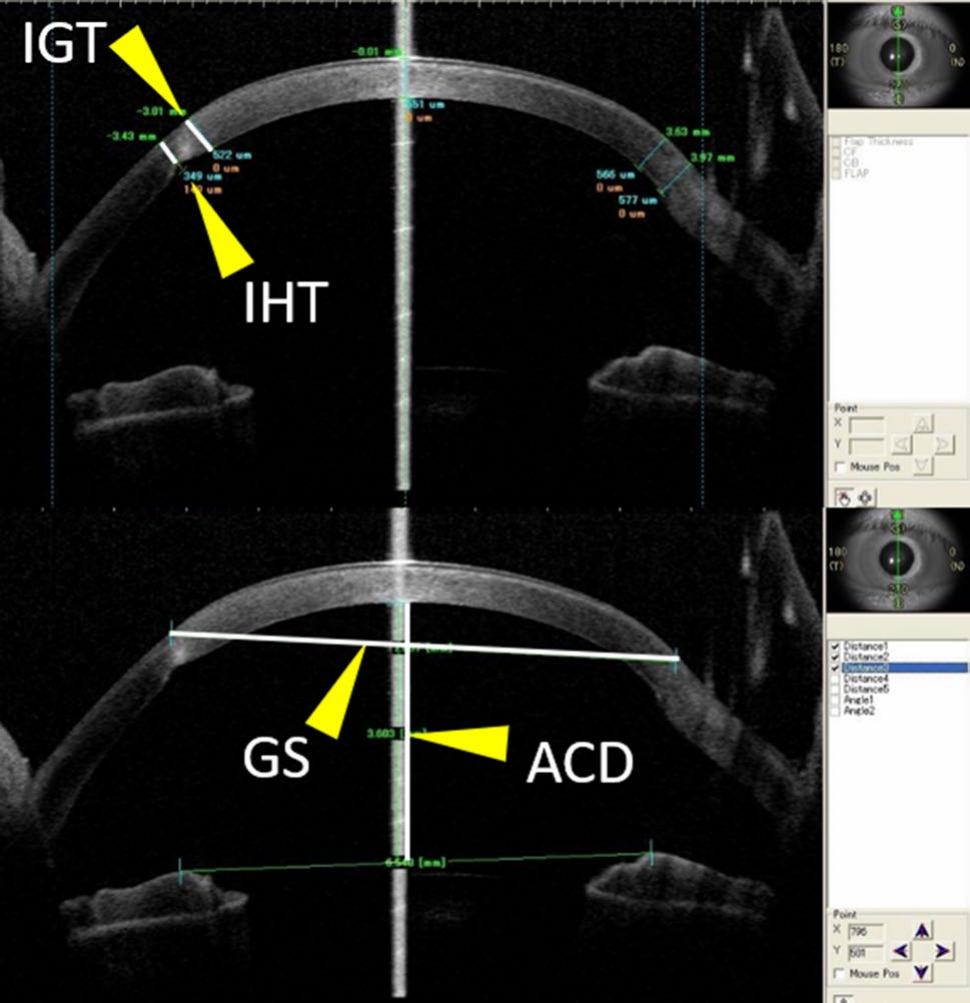
Scheme of each parameter measured by anterior segment optical coherence tomography. Vertical images were used for measurements. The thicknesses of the thinnest parts of the inferior graft (IGT) and host (IHT) cornea, graft size (GS), and anterior chamber depth (ACD) were measured.

Subjects in this study were recruited from among those who visited the University of Tokyo Hospital Cornea clinic between December 2015 and February 2016. Seventy-six patients who had maintained clear grafts for more than 10 years after PK and had undergone AS-OCT (CASIA, SS-1000/SS-2000, Tomey, Japan) were enrolled in this study. The patients were divided into 2 groups according to the primary indication for PK: KC or Others groups. Patient age at PK (age at PK), number of years after PK when AS-OCT was performed (postoperative period), current age and sex were recorded. Graft size, ACD, CCT, IHT, and IGT were measured from AS-OCT vertical images (Fig. 4). The ratios of IHT and IGT to CCT (IHT/CCT and IGT/CCT) were calculated as the inferior corneal thinning indicators. ACD was used to evaluate corneal protrusion. In the present article, we refer to IHT/CCT, IGT/CCT, and ACD as corneal ectasia indicators. Keratometric parameters, such as Ks, AvgK, CYL, Fourier analysis parameters (Sph, Reg, Asym and HOI), and K_max_ were also obtained from AS-OCT. The data were compared between the 2 groups. Correlations were examined between each parameter except between the keratometric parameters in both groups. Multivariate regression analyses were performed in each group to determine the risk factors associated with corneal ectatic change and to determine the factors associated with keratometric changes. To determine corneal ectasia risk factors, 5 possible explanatory variables (age at PK, current age, postoperative period, sex, and graft size) were evaluated with the objectives of corneal ectasia indicators (IHT/CCT, IGT/CCT, and ACD). To determine the factors associated with keratometric changes, the 3 explanatory variables of corneal ectasia indicators (IHT/CCT, IGT/CCT, and ACD) were examined with the objectives of the AS-OCT keratometric parameters (Ks, AvgK, CYL, Sph, Reg, Asym, and K_max_).

### Statistical analysis

Data comparison was performed by t-test, except the man to woman ratio, which was analyzed by Fisher’s exact test. Pearson correlation coefficients were obtained for the correlation analysis. A parameter combination with minimum corrected AIC was selected by the stepwise method to determine the best model for each multivariate regression analysis. A *p* < 0.05 was considered statistically significant. The statistical analyses were performed using JMP Pro 13 (SAS Institute, Cary, NC, USA).
